# Tropical/Subtropical Peatland Development and Global CH_4_ during the Last Glaciation

**DOI:** 10.1038/srep30431

**Published:** 2016-07-28

**Authors:** Hai Xu, Jianghu Lan, Enguo Sheng, Yong Liu, Bin Liu, Keke Yu, Yuanda Ye, Peng Cheng, Xiaoke Qiang, Fengyan Lu, Xulong Wang

**Affiliations:** 1 State key Laboratory of Loess and Quaternary Geology, institute of Earth Environment, Chinese Academy of Sciences. Xi’an, China; 2 Department of Environment Science and Technology, School of Human Settlements and Civil Engineering, Xi’an Jiaotong University, Xi’an, China; 3 College of Tourism and Geographical Sciences, Yunnan Normal University, Kunming, China; 4 Graduate university of Chinese Academy of Sciences. Beijing, China

## Abstract

Knowledge of peatland development over the tropical/subtropical zone during the last glaciation is critical for understanding the glacial global methane cycle. Here we present a well-dated ‘peat deposit-lake sediment’ alternate sequence at Tengchong, southwestern China, and discuss the peatland development and its linkage to the global glacial methane cycle. Peat layers were formed during the cold Marine Isotope Stage (MIS)-2 and -4, whereas lake sediments coincided with the relatively warm MIS-3, which is possibly related to the orbital/suborbital variations in both temperature and Asian summer monsoon intensity. The Tengchong peatland formation pattern is broadly synchronous with those over subtropical southern China and other tropical/subtropical areas, but it is clearly in contrast to those over the mid-high Northern Hemisphere. The results of this work suggest that the shifts of peatland development between the tropical/subtropical zone and mid-high Northern Hemisphere may have played important roles in the glacial/interglacial global atmospheric CH_4_ cycles.

Ice core records indicate that the global methane concentration varied rapidly and periodically on orbital/suborbital timescales^1^,^2^. Wetlands are the largest single natural source of CH_4_ emissions, representing 20% to 40% of the total CH_4_ emissions budget^3^,^4^; and the fluctuations in wetlands CH_4_ emissions could explain 70% of the substantial inter-annual anomalies in atmospheric CH_4_ concentrations^5^. Peatlands in the high latitudes of the Northern Hemisphere have long been regarded as the most important source of global wetlands methane emission^6^,^7^,^8^. Tropical/subtropical peat burial is estimated to be ~10% of that in the high latitude Northern Hemisphere^9^,^10^,^11^,^12^, and therefore the tropical/subtropical peatlands have been deemed to be less important in the global methane cycle. However, during the past approximately two decades, a large number of modern observations^13^ and simulations^3^,^4^,^5^,^14^ show much higher methane emission fluxes from tropical/subtropical wetlands, and therefore largely challenge the predominating role of the northern peatlands in global methane cycle^4^. For example, Bloom *et al.*^3^ suggested that tropical wetlands may contribute 52 to 58% of global emissions, with the remainder coming from the extra-tropics. Bridgham *et al.*^4^ reviewed atmospheric inversion studies and show that about 47 to 89% (median 73%) of global wetland CH_4_ emissions is originated from tropical wetlands due to their large areal extent and high CH_4_ fluxes.

Then how is the relative contribution of wetlands methane emission from tropical/subtropical zone as compared to those from the northern high hemisphere during the glacial stadial periods (cold phases in glacial times)? During the cold phases of the last glaciation, because of the significant decreases in temperature, wetland biomass in the high latitude Northern Hemisphere was sharply reduced, and methane release from the northern peatlands could also be largely decreased. This suggests that wetlands in other areas, such as the tropical/subtropical zone, could have an increasing contribution to the global methane cycle during the last glacial stadial periods. A large number of peatland initial ages suggest that during the switch from the last glaciation to the Holocene, i.e., “last deglaciation~ Bølling and Allerød (B/A) warm interval~ Younger Dryas~ Preboreal”, peatlands extension in the high latitude Northern Hemisphere remained relatively steady, whereas the global atmospheric methane concentration varied dramatically^8^. This also implies that a potential important atmospheric methane source existed on glacial/interglacial timescales, and its relative contribution varied significantly with climate changes on similar timescales. The tropical/subtropical peatlands are likely to be such a potential candidate in the glacial/interglacial global methane cycles^15^. The δ^13^C values of atmospheric methane in Antarctic ice cores showed large differences between glacial stadial and interstadial periods, which could not be simply explained by temperature variations; rather, the different values pointed to the possibility that tropical wetland methane emissions could have accounted for a higher fraction during the stadial periods than during the interstadial periods^16^,^17^. Therefore, peatland development in tropical/subtropical areas and its contribution to global atmospheric methane concentrations have received increasing attention over the past few decades^3^,^4^,^5^,^10^,^13^,^14^. Here we report peatland development during the last glaciation at Tengchong, southwestern China, and discuss the possible linkages to orbital scale climatic changes and global methane concentrations.

## Background, Sampling, and Methods

**Figure 1:**
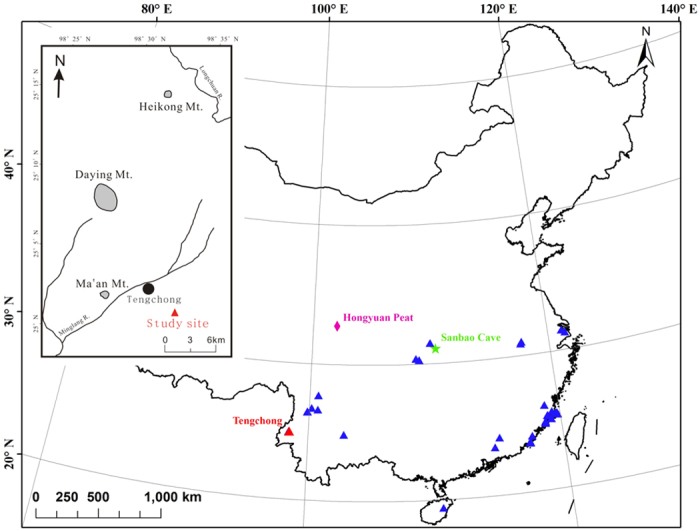
Location of the Tengchong peatland (red triangle). Also shown are the locations of Sanbao cave (green star) and Hongyuan peatlands (magenta diamond), and the locations of peatlands originated during the MIS-2 period in subtropical China (blue triangles; data from Zhao *et al.*^19^; generated in ArcGIS 10; http://www.esri.com/). Note parts of the peatland sites are overlapped, for details please refer to Zhao *et al.*^19^. The upper left panel shows the volcanic eruption sites of Mt. Ma’an, Mt. Daying, and Mt. Heikong (grey shades)^20^,^21^.

Tengchong County is located in Yunnan province, southwestern China (Fig. 1). Precipitation here is dominated by the Indian summer monsoon^18^. The mean annual temperature is ~15.1 °C, with mean January and August temperatures of ~8.1 °C and ~19.9 °C, respectively. The mean annual precipitation is ~1527 mm, ~80% of which occurs from May to October (1971–2000 AD, climate data from Tengchong weather station, China Meteorological Data Sharing Service Platform, http://cdc.cma.gov.cn).

**Figure 2:**
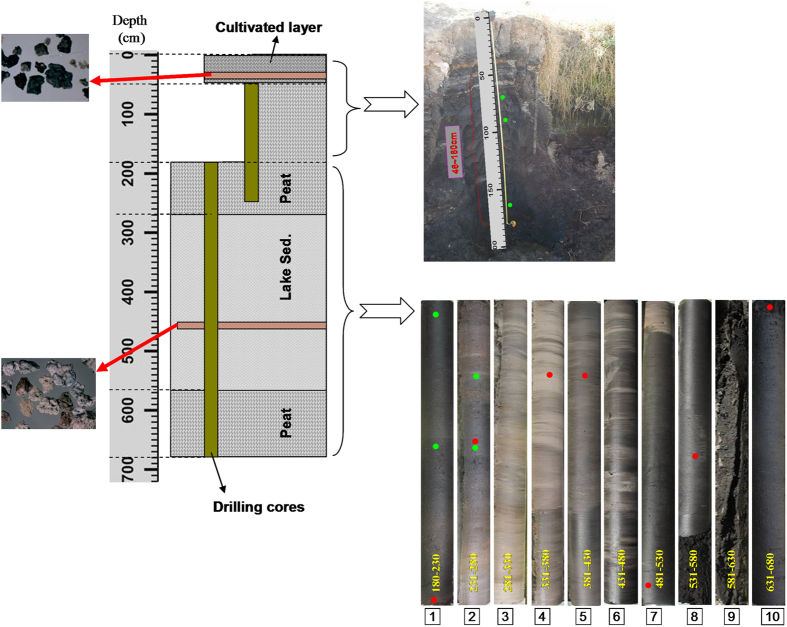
Profile of core TC-11–1. The upper 1.8 m is collected from an outcrop, whereas the lower parts are drilling cores. The left two pictures show the microphotograph of volcanic debris in two volcanic layers at 40 cm and 465 cm. The right two pictures show the photograph of the outcrop and drilling cores, respectively (see large Figures in Supplementary Figures). Green and red circles show AMS^14^C dating for cellulose and bulk organic matter, respectively (see details in Table S1).

A 6.8 m core (TC-11–1; 25°0′37″N, 98°31′26″E; Alt. 1630 m) was obtained on December, 2011 (Figs 2 and S1). The upper most 0–46 cm of core TC-11–1 is a modern cultivated clay layer (Fig. 2), and the closely underlain layer is a peat layer (47–270 cm; Fig. 2). A thick lacustrine clay layer (271–570 cm) is below the peat layer, and below this lacustrine sediment section is another peat layer (bottom not reached; Figs 2 and S1). Two parallel cores (TC-14–1 & 2) were obtained at the same site in 2014, and the sedimentary facies of these cores are well correlated with those of core TC-11–1 (Fig. S2).

**Figure 3:**
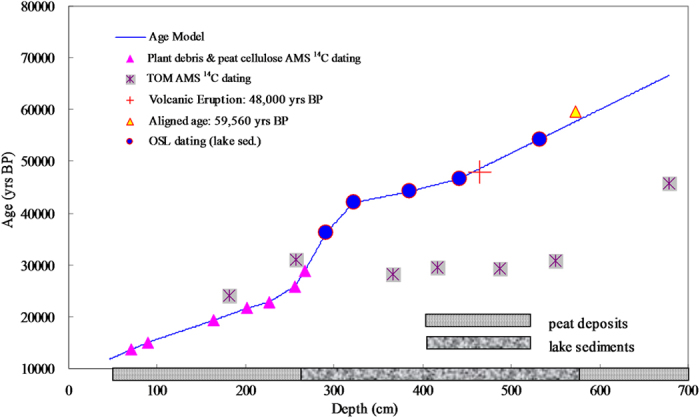
Geochronology of core TC11–1. The age model is obtained from the interpolation between ^14^C ages (filled pink triangles) of plant and cellulose samples of the upper peat section (47–270 cm; Table S1) and the OSL ages (filled blue circles) of the middle lacustrine sediment section (Table S2). Grey cross squares show the ^14^C ages based on total organic matter (not used in the age model; see text). The cross symbol (red) denotes the volcanic eruption age at surrounding areas (~46,000–50,000 BP)^20^,^21^. The yellow filled triangle shows the transition age from MIS-4 to MIS-3 inferred from the speleothem δ^18^O curve at Sanbao Cave (~59,560 BP)^24^. Horizontal filled columns show the lithology of core TC-11–1.

Core TC-11–1 was sampled at 1-cm intervals. Magnetic susceptibility (MS) was determined on a Bartington MS2C, and other indices, including bulk density, humification, and total organic carbon content (TOC), were also determined (see methods in ref. 22). Radiocarbon dating for the peat section of core TC-11–1 was performed based on plant debris and cellulose (extracted from peat deposits; see method in ref. 23; Table S1). The lacustrine section (271–570 cm) of core TC-11–1 was also dated by ^14^C dating based on total organic matter (TOM; Table S1; Fig. 3). Optically Stimulated Luminescence (OSL) dating was performed based on fine-grained (4–11 μm) quartz in sediments of the lacustrine section of core TC-14–2 (Table S2; Fig. S3).

### Geochronology

The ^14^C ages of the upper peat section (47–270 cm) exhibit a good stratigraphic sequence, and the ^14^C ages of plant debris are similar to those of the cellulose samples (Table S1; Fig. 3). A thin volcanic layer was clearly observed at ~40 cm (core TC-11–1; Figs 2 and S1), and previous ^14^C dating showed that this volcanic layer was deposited around the Younger Dryas (*Liu et al.*, *unpublished results*). The calibrated ^14^C age at 46 cm of core TC-11–1 is ~11,570 BP, which is close to the age of the volcanic layer. However, the TOM ^14^C ages are relatively constant (varied around 30,000 BP; Fig. 3), and the ^14^C age of the bottom peat section is >45,000 BP (Table S1). These suggest that the ^14^C dating (based on bulk organic carbon) is limited for lake sediments older than 30,000 BP in this case. The OSL dating results show that the lacustrine sediments were deposited during MIS-3 (Table S2; Fig. 3). We therefore use the ^14^C ages of the upper peat section (47–270 cm) and the OSL ages of the lacustrine section to establish a combined age model for core TC-11–1, whereas the TOM ^14^C ages of the lacustrine section were not used (Fig. 3).

**Figure 4:**
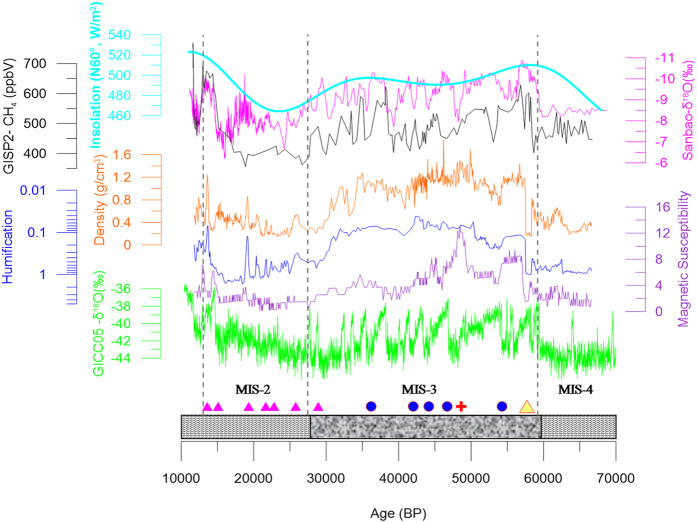
Indices in core TC-11–1, including bulk density (orange), humification (blue), and magnetic susceptibility (purple). Also shown are the solar insolation at 60° N latitude (sky blue)^27^, the GISP2 CH_4_ concentration (black)^1^, the Sanbao cave δ^18^O (pink)^24^, and the Greenland ice core δ^18^O (GICC05; green)^28^. Pink triangles and blue filled circles show the date-controlling points of the Tengchong profile (similar to Fig. 3). The cross symbol (red) and filled triangle (yellow) show the volcanic eruption age and the transition age from MIS-4 to MIS-3 (inferred from ref. 24), respectively. The horizontal filled columns show the lithology of core TC-11–1. The vertical dashed lines broadly show the boundaries of Holocene/MIS-2, MIS-2/MIS-3, and MIS-3/MIS-4.

An abnormal increase in magnetic susceptibility was found at 465 cm in core TC-11–1 (Figs 4 and S4), and volcanic debris were identified under a microscope (Fig. 2). Previous studies indicated volcanic activities during the period of ~46,000–50,000 BP at adjacent sites, such as Mt. Heikong, Mt. Daying, and Mt. Ma’an^20^,^21^ (Fig. 1). The age for the volcanic layer at 465 cm in core TC-11–1 is ~48,000 BP (Fig. 4), which is within the age range of the adjacent volcanic eruptions, suggesting that the volcanic debris in core TC-11–1 may come from the adjacent volcanic eruption. Therefore, this volcanic layer (at 465 cm) can serve as an evidence to support the age model of core TC-11–1. Based on this age model, the upper peat section was formed during MIS-2, whereas the lacustrine sediment section was deposited during MIS-3. The ^14^C age of the ‘upper peat section-lake sediment’ transition point was ~27,000 BP (Fig. S4), which is similar to the age of the transition from MIS-3 to MIS-2^24^,^25^. The bottom peat layer was deposited during MIS-4, and the age of the transition point from the bottom peat layer to the upper lake sediment section was ~57,550 BP (Fig. S4), which is also close to the age of the transition point from MIS-4 to MIS-3 as inferred from the speleothem δ^18^O curve (Fig. 3; ~59,560 BP^24^), further supporting the reliability of the age model.

### Tengchong peatland development

Our previous work showed that TOC, bulk density, and humification are important indices to indicate peatland development across the Eastern margin of the Tibetan Plateau (ETP)^22^,^26^. Higher peatland formation rates lead to higher TOC and humification^22^,^26^, which is also true in the records of the Tengchong peat deposits (Fig. S5). When organic matter accounts for a higher fraction, the proportion of terrestrial clastic debris is lower, resulting in lower bulk density and MS values^22^ (Figs 4 and S5). On the other hand, lower peatland formation rates correspond to lower TOC values, lower humification, but higher bulk density and higher MS values^22^.

It was cold and dry during MIS-2 and MIS-4, but relatively warm and wet during MIS-3, as inferred from the Greenland ice core δ^18^O^28^ and the speleothem δ^18^O curves, respectively^24^ (Fig. 4). The geochronology of core TC-11–1 showed that the peat layers were formed during the cold MIS-2 and MIS-4 periods. During the relatively warm MIS-3, Tengchong peat deposition was disrupted, and the wetland was submerged and converted to a lake. Such a peatland development pattern is possibly a response to orbital scale variations in both Asian summer monsoon precipitation and global temperatures, which are closely related to changes in solar insolation^2^,^18^,^24^,^27^,^29^.

The Tengchong peatland development pattern suggested that although global temperatures decreased during the MIS-2 and MIS-4 periods, they were still favorable for wetland development at Tengchong due to the high background temperature. Decreased temperatures did not obviously reduce the wetland biomass; rather, the cooler temperatures favored peat deposition due to decreased organic matter decomposition rates under cooler conditions. In addition, lower temperatures led to higher soil moisture contents due to lower evaporation, which resulted in lower oxygen concentrations in peatland pores and, thereafter, lower organic matter degradation rates^22^,^26^,^30^,^31^. During the warm Holocene, although plant biomass could be higher, the increased temperature also accelerated the organic matter decomposition rate. Higher temperature may also lead to stronger evaporation and, consequently, lower soil moisture content, which is unfavorable for organic matter preservation^22^,^26^,^30^,^31^.

### Comparison of Tengchong peatland formation patterns with others

The peatland formation pattern at Tengchong basin is in contrast to those in the mid and northern ETP areas^26^. For example, at the Zoige Plateau, mid ETP area, and at Qilian Mt., northern ETP area, peatlands of the MIS-2 and MIS-4 periods were mostly non-existent, which could be ascribed to the low plant biomass due to the cold climatic conditions during these cold periods. However, during the warm Holocene, peat deposition was widespread over the northern, mid, and southern ETP areas^22^,^26^,^32^,^33^. The peatland formation pattern at Tengchong basin is also in contrast to those of the high latitude Northern Hemisphere. For example, peatlands were extensive from the last deglaciation to the Holocene over Siberia, the circum-Arctic Ocean areas, Alaska areas, and north Canada, etc.^6^,^7^,^8^,^10^; however, these areas could have been covered by permafrost during the previous cold stadial periods^34^, such as MIS-2, and peatlands could have been much less extensive.

The Tengchong peatland formation pattern is similar to those over subtropical China. For example, during the cold MIS-2 period, a large number of peatlands were formed in subtropical China, including in the Fujian province, Hainan province, and Guangdong province^19^,^35^,^36^ (Fig. 1). The Tengchong peatland formation pattern is also broadly similar to those in tropical/subtropical areas^10^,^11^. Therefore, the differences in peatland formation patterns between Tengchong and mid and northern ETP areas exemplify the different peatland formation patterns between tropical/subtropical areas and the mid-/high latitudes of the Northern Hemisphere.

### Tropical/subtropical wetlands methane emission and global methane

Wetland methane emission fluxes are influenced by variable factors, including soil temperature, soil water table, vegetation types, mineral composition, biological communities, etc. Generally, there is no simple relationship between these controlling factors and methane emissions; the wetland methane emission simulations should also be considered separately according to different wetland sites^37^,^38^. Harriss *et al.*^39^ reported that methane emission flux of wetlands over northern United States vary between 0.003 and 1.94 g CH_4_ m^−2^ day^−1^, with half of these values between 0.1 and 0.4 g CH_4_ m^−2^day^−1^, and an average value of 0.337 g CH_4_ m^−2^day^−1^. On the other side, the observed and simulated wetlands methane emission fluxes over the tropical/subtropical zone are much higher than those over the northern high hemisphere, which may be related to the higher wetlands biomass due to higher temperature and precipitation. The higher wetlands methane emission fluxes over the tropical/subtropical zone may also partly due to the higher fraction of mineral soil wetlands, which have much higher methane fluxes as compared with those over northern high hemisphere^4^,^40^. Therefore, tropical/subtropical wetlands could have played an important role in the global atmospheric methane cycle. For example, atmospheric inversion studies estimate that from 47 to 89% (median 73%) of global wetland CH_4_ emissions may originate from tropical wetlands^4^. Bloom *et al.* also suggested that tropical wetlands contribute 52 to 58% of global emissions, with the remainder coming from the extra-tropics^3^.

On long term timescales, because most parameters remain relatively constant, whereas the soil temperature, soil water table, as well as soil biological communities are linked to climatic changes, it seems that the factors that influence climatic changes, e.g., the atmospheric circulations, monsoon intensity, etc., should also modulate the long term variations in wetlands methane emissions. Based on satellite observations, Bloom *et al.*^3^ showed that both the spatial and temporal wetland methane emission fluxes over tropical/subtropical zone are mainly positively correlated to the equivalent groundwater depth, but negatively/weakly correlated to surface skin temperatures. This finding also implies that the long term variations in effective precipitation could be critical in modulating the tropical/subtropical methane emission fluxes. On long term timescales, higher effective precipitation corresponds to higher soil water table, which leads to higher wetland biomass, higher activities of the anaerobic methane bacteria, and higher CH_4_ emission flux, and vice versa. Hodson *et al.*^41^ simulated wetland methane emissions based on a simple modeling approach and examined the relationship between modeled methane emission flux and El Nino-Southern Oscillation (ENSO) index; the results suggest that inter-annual variability in CH_4_ emissions from wetlands is strongly influenced by the ENSO cycle, with 44% of the inter-annual variability in CH_4_ emissions from tropical wetlands explained by ENSO. A three years observation shows that the peatland methane emission flux at the Zoige Plateau, northeastern Tibetan Plateau, is closely correlated to changes in standing water depths during the growing season^42^, suggesting a dominant role of precipitation in controlling annual/inter-annual peatland methane emission flux. A similar relationship between paleo- precipitation and peatlands methanogenesis at Hongyuan (see location in Fig. 1), the Zoige Plateau, was also reported by Zheng *et al.*^43^. These lines of evidence suggest that the historical CH_4_ emissions could be closely related to the paleoclimatic changes.

With respect to the Tengchong peatland, although we have no idea of the CH_4_ emission flux during the glacial times, it is reasonable that the obvious decreases in temperatures during the glacial stadial periods could have led to higher effective precipitation and thereafter higher methane emission fluxes. As the Tengchong peatland was better developed during the cold periods (Fig. 4), it is likely that they could have released more CH_4_ to the atmosphere during the glacial stadial periods.

As mentioned above, during the MIS-2 period, a large number of peatlands were formed near Chinese coastal areas^19^,^35^,^36^ (Fig. 1). Moreover, the coastal areas could have been much larger during the stadial period (as compared to modern areas) due to the decreased sea level. For example, the sea level at the Eastern China Sea and Southern China Sea was ~130–150 m and 100–120 m lower than the modern sea level, respectively^44^. The coastal line moved ~1000 km eastward of the modern coastal line, and the area of the China Sea was reduced by approximately 1/3 during the glacial period^44^. From a global perspective, the global mean sea level decreased ~120 m during the glacial period^45^, indicating that a considerably larger tropical/subtropical land area could have emerged during glacial times. Because of the favorable thermo- and hydro-climatic conditions, peatlands over the tropical/subtropical zone could have covered a considerably larger area. Due to the relatively low elevation of southern and eastern Asia, most of the tropical/subtropical peat burials occurred in these areas (more than 70%)^10^,^11^,^12^, therefore, it is reasonable that the subtropical/tropical southern to eastern Asia could have played a more important role in the global glacial methane cycles. During the transition from glacial stadial to interstadial periods (such as from MIS-4 to MIS-3), because of the switch from inland peatlands to lakes (e.g., the Tengchong peatland) and the switch of coastal peatlands to ocean (due to the increases in sea levels) over the tropical/subtropical areas, higher methane emission fluxes could be imaged from these peatlands during these transitional periods due to the increased anaerobic microbiological activities (under deeper submerged conditions), which may partly contribute to the sharp increases in global atmospheric methane concentration during the transitional periods (such as from MIS-4 to MIS-3; Fig. 4). In addition, because methane has a strong infrared radiation absorbing effect (approximately 25 times higher than that of CO_2_)^46^, the tropical/subtropical methane released during the glacial periods may have played an important role in buffering the global glacial temperature decreases.

## Additional Information

**How to cite this article**: Xu, H. *et al.* Tropical/Subtropical Peatland Development and Global CH_4_ during the Last Glaciation. *Sci. Rep.* **6**, 30431; doi: 10.1038/srep30431 (2016).

## Supplementary Material

Supplementary Information
