# Supplementary material for: Tropical/Subtropical Peatland Development and Global CH4 during the Last Glaciation
Source: Sci Rep. 2016 Jul 28;6:30431. doi: 10.1038/srep30431 (PMC4964359; doi:10.1038/srep30431)
Supplement: Supplementary Information [file srep30431-s1.pdf]

# Tropical/Subtropical Peatland Development and Global $CH_4$ during the Last Glaciation

**Hai Xu [1, 2], Jianghu Lan [1], Enguo Sheng [1], Yong Liu [3], Bin Liu [1], Keke Yu [1, 4], Yuanda Ye [1, 4], Peng Cheng [1], Xiaoke Qiang [1], Fengyan Lu [1], Xulong Wang [1]**

1. State key Laboratory of Loess and Quaternary Geology, institute of Earth Environment, Chinese Academy of Sciences. Xi'an, China.
2. Department of Environment Science and Technology, School of Human Settlements and Civil Engineering, Xi'an Jiaotong University, Xi'an, China.
3. College of Tourism and Geographical Sciences, Yunnan Normal University, Kunming, China
4. Graduate university of Chinese Academy of Sciences. Beijing, China.

Corresponding author: Hai XU (xuhai@ieecas.cn)

**Address:**

Yanxiang Road, #97, Xi'an, Shaanxi province, China

Post Code: 710061

Tel: 86-29-62336295

Mobile: 86-0-13991378151

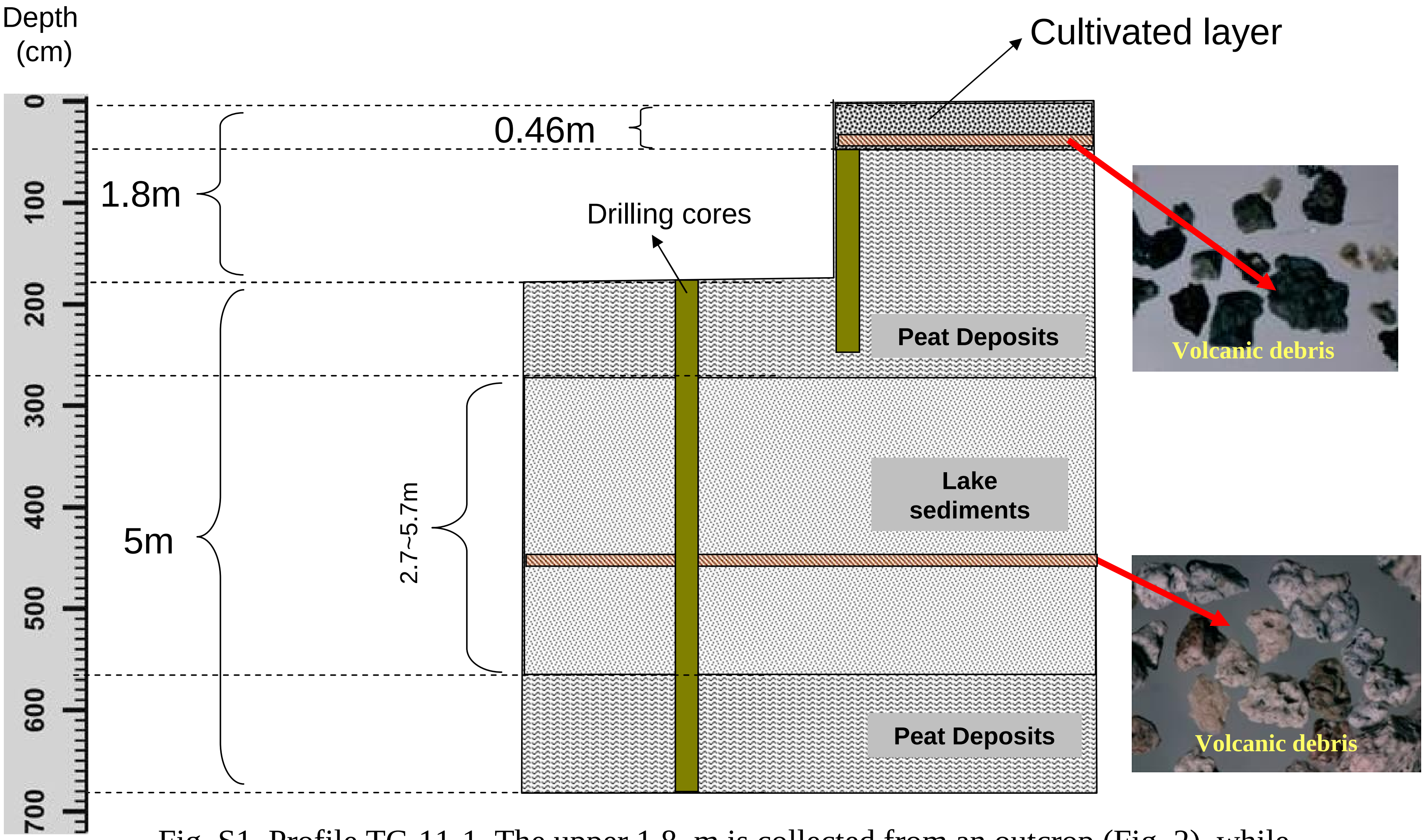


Fig. S1. Profile TC-11-1. The upper 1.8 m is collected from an outcrop (Fig. 2), while the lower parts are drilling cores (Fig. 2). Two volcanic layers were identified at 40 cm and 465 cm, respectively.

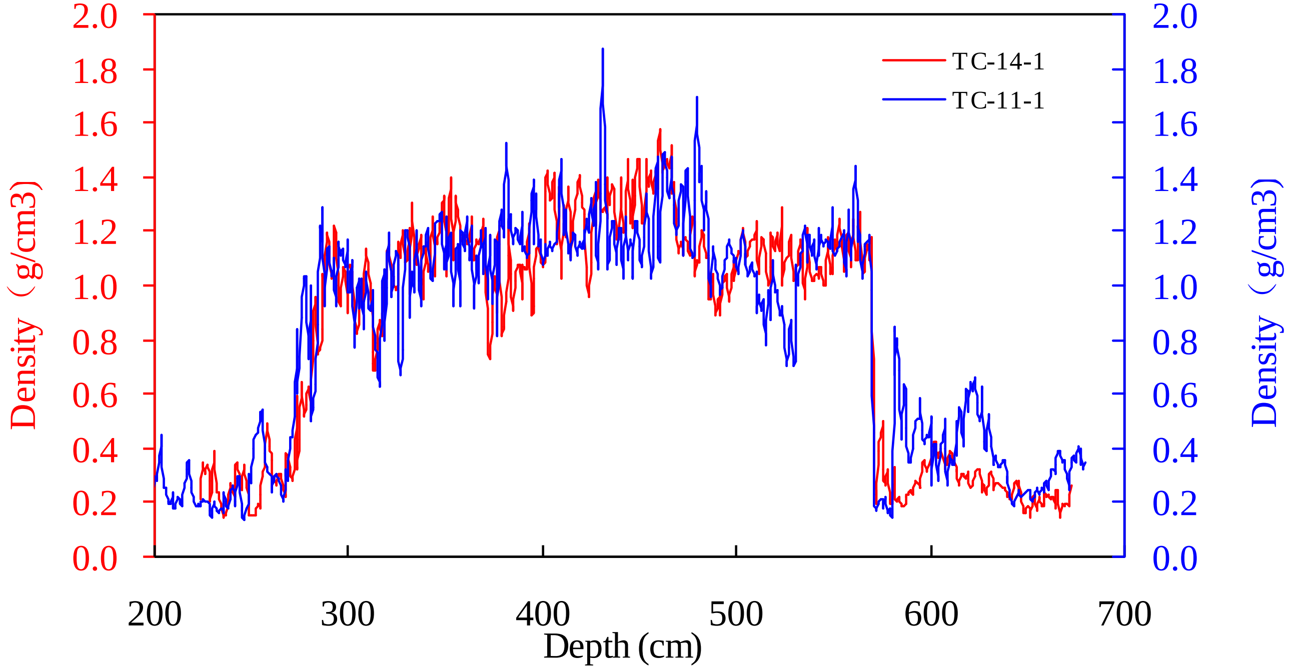


Fig. S2. Stratigraphic comparison of the densities of core TC-11-1 and core TC-14-1.

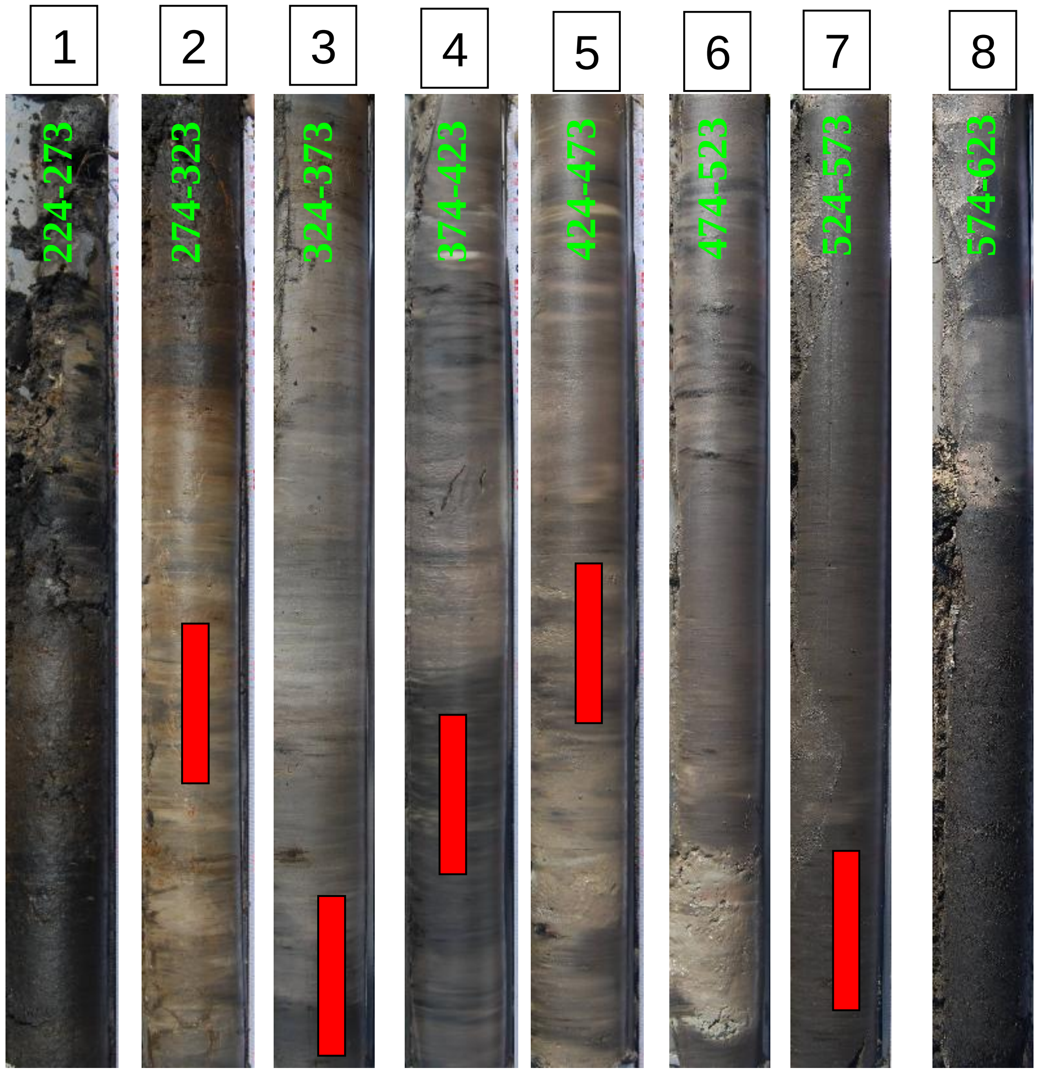


Fig. S3. Photography of core TC-14-2 (224-623cm).
Red columns show OSL dating samples  (see details in Table S2).

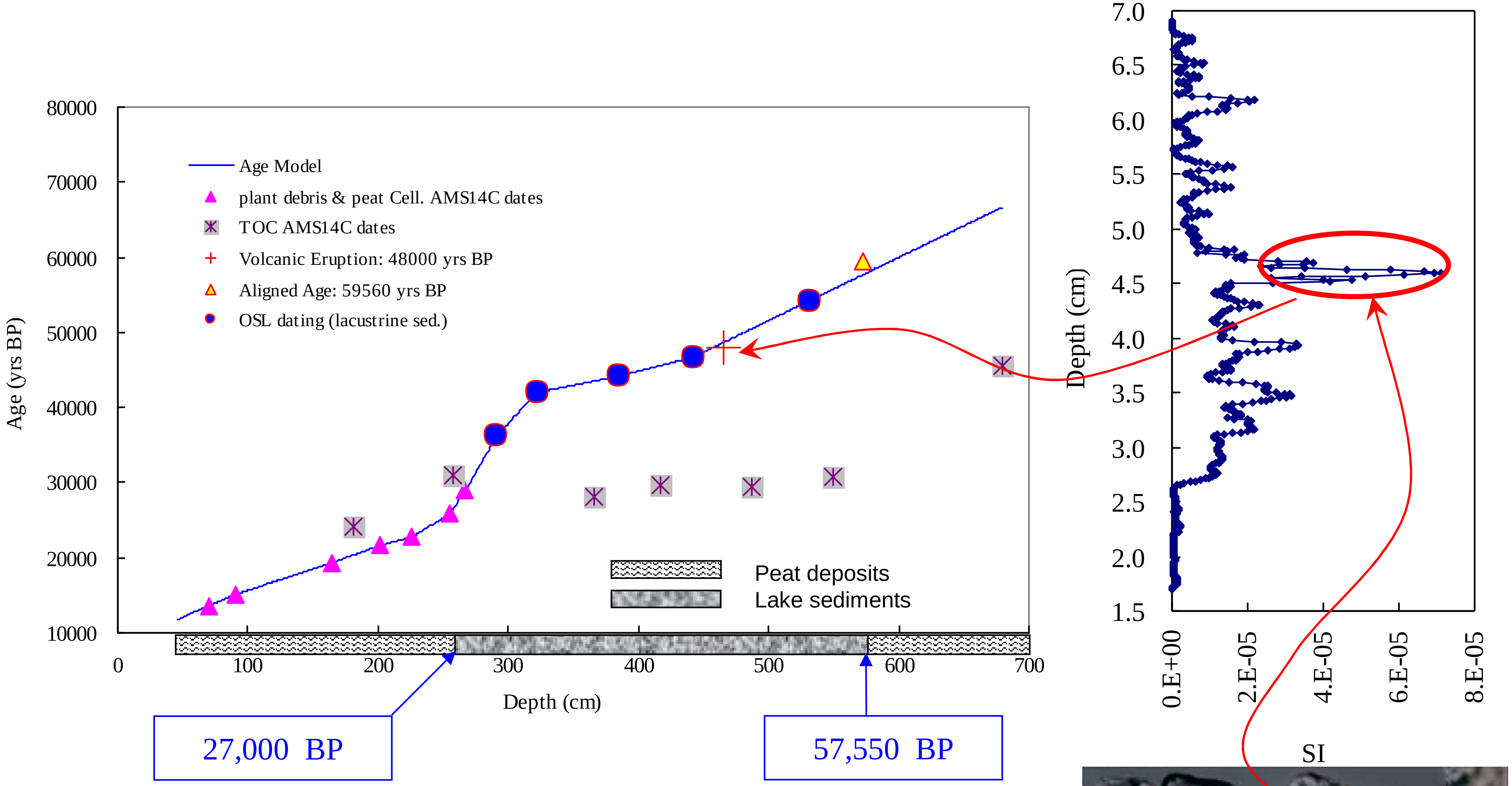


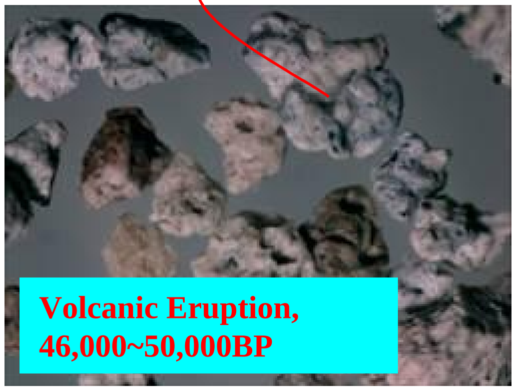


Fig. S4. Left panel shows the age model of profile TC-11-1, and the ages of two transition points between peat and lake sediment sections. Right upper panel shows magnetic susceptibility of TC-11-1, and right lower panel shows micrograph of the volcanic materials in the lower volcanic layer.

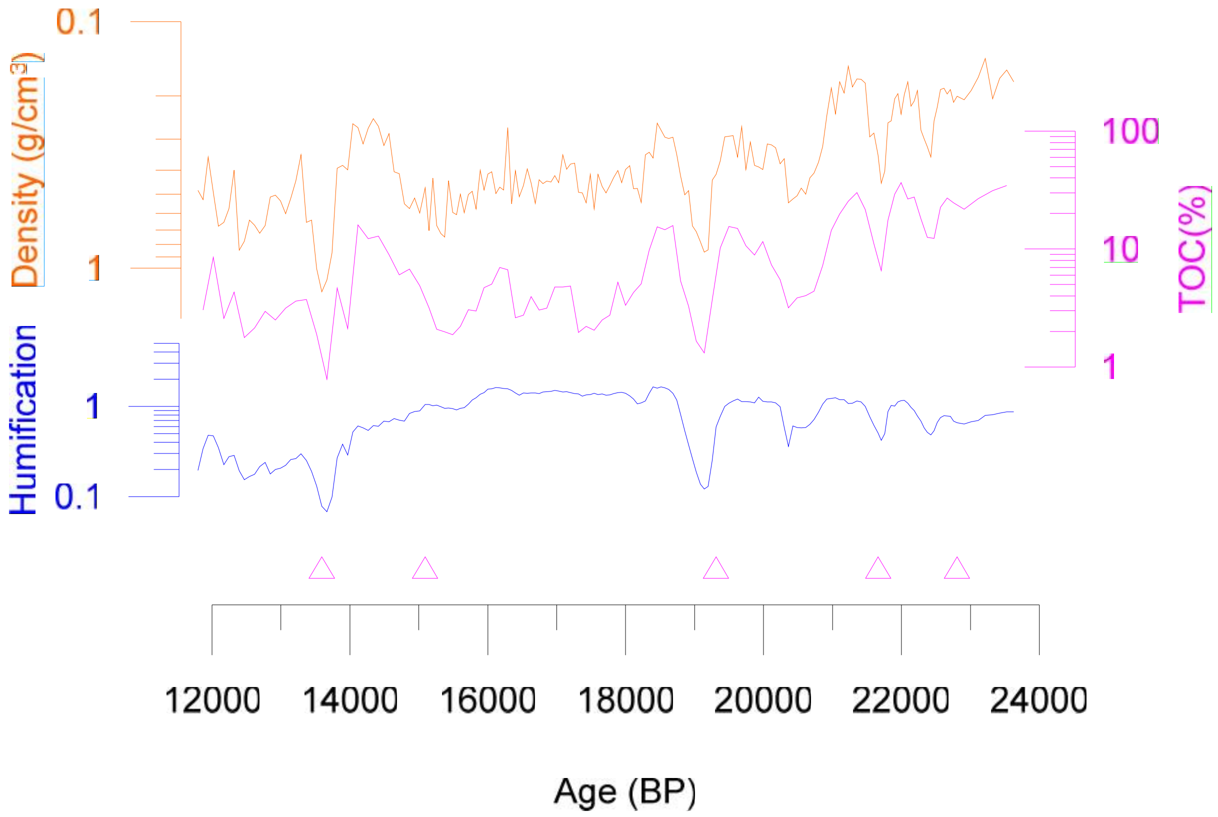


Fig. S5. TOC, density, and humification of the upper peat section (46-270cm) in core TC-11-1. Pink triangles represent the dating points.

**Table S1**. $^{14}C$ ages of samples in core TC-11-1

| Dating materials | Lab Code | Sample Code | Depth (cm) | $^{14}C$ age | Corrected error | Calib. $^{14}C$ ages (median prob.) * |
|---|---|---|---|---|---|---|
| Plant debris/cellulose | XA6629 | YB70 | 70 | 11747 | 46 | 13593 |
| | XA6636 | YB90 | 90 | 12725 | 45 | 15090 |
| | XA7767 | TC11-1-164 | 164 | 16151 | 108 | 19311 |
| | XA7777 | TC11-1-202 | 202 | 18152 | 108 | 21661 |
| | XA7762 | TC11-1-226 | 226 | 19133 | 112 | 22805 |
| | XA7764 | TC11-1-255 | 255 | 21532 | 136 | 25790 |
| | XA7765 | TC11-1-267 | 267 | 24072 | 188 | 28898 |
| Bulk organic matter | XA6917 | TC11-1-181 | 181 | 20162 | 75 | 24091 |
| | XA6890 | TC11-1-257 | 257 | 26440 | 107 | 31073 |
| | XA6889 | TC11-1-366 | 366 | 23365 | 78 | 28192 |
| | XA6915 | TC11-1-417 | 417 | 24777 | 99 | 29594 |
| | XA6916 | TC11-1-487 | 487 | 24451 | 91 | 29338 |
| | XA6919 | TC11-1-550 | 550 | 26074 | 111 | 30835 |
| | XA6911 | TC11-1-679 | 679 | 42457 | 343 | 45658 |

* $^{14}C$ ages were calibrated by software of Calib 6.02.[1]

**Table S2**. Optically Stimulated Luminescence dating results for lake sediments in core TC-14-2.

| Sample No. | Depth (cm) | U (ppm) | Th (ppm) | K (%) | Water contents（%） | Dose rate (Gy/ka) | | Equivalent Dose (Gy) | | Age (Ka) | |
|---|---|---|---|---|---|---|---|---|---|---|---|
| XH140716-6 | 291 | 5.49 | 65.42 | 1.59 | 43±20 | 6.96 | ±0.57 | 251.94 | ±9.61 | 36.21 | ±3.27 |
| XH140716-7 | 322 | 5.34 | 65.54 | 1.52 | 40±20 | 7.05 | ±0.59 | 296.04 | ±10.02 | 42.02 | ±3.81 |
| XH140716-8 | 385 | 4.97 | 60.7 | 1.18 | 32±15 | 6.81 | ±0.51 | 300.8 | ±9.52 | 44.16 | ±3.59 |
| XH140716-9 | 442 | 4.65 | 54.04 | 1.62 | 30±15 | 6.70 | ±0.48 | 312.79 | ±9.93 | 46.70 | ±3.68 |
| XH140716-10 | 532 | 4.59 | 53.15 | 1.44 | 77.8±10 | 4.54 | ±0.24 | 246.59 | ±6.72 | 54.27 | ±3.19 |

References


1. Stuiver, M. *et al.* INTCAL98 radiocarbon age calibration, 24,000-0 cal BP. *Radiocarbon* **40,** 1041-1083 (1998).
